# CD90^low^ MSCs modulate intratumoral immunity to confer antitumor activity in a mouse model of ovarian cancer

**DOI:** 10.18632/oncotarget.27065

**Published:** 2019-07-09

**Authors:** Yang Zeng, Binghao Li, Tao Li, Wei Liu, Chongzhao Ran, Richard T. Penson, Mark C. Poznansky, Yanan Du, Huabiao Chen

**Affiliations:** ^1^Vaccine and Immunotherapy Center, Massachusetts General Hospital and Harvard Medical School, Boston 02114, USA; ^2^Department of Biomedical Engineering, School of Medicine, Collaborative Innovation Center for Diagnosis and Treatment of Infectious Diseases, Tsinghua University, Beijing 100084, China; ^3^Martinos Center for Biomedical Imaging, Department of Radiology, Massachusetts General Hospital and Harvard Medical School, Charlestown 02129, USA; ^4^Medical Gynecologic Oncology, Gillette Center for Women's Cancers, Massachusetts General Hospital and Harvard Medical School, Boston 02114, USA; ^5^Department of Cancer Biology, Dana Farber Cancer Institute, Boston 02215, USA; ^6^Department of Orthopaedics, The Second Affiliated Hospital of Zhejiang University School of Medicine, Hangzhou, Zhejiang 310009, China; ^7^Jiangsu Key Laboratory of Clinical Laboratory Medicine, School of Medicine, Jiangsu University, Zhenjiang, Jiangsu 212013, China

**Keywords:** mesenchymal stem cells, CD90, ovarian cancer, immunotherapy

## Abstract

Both anti-tumoral and pro-tumoral effects of mesenchymal stem cells (MSCs) in preclinical treatment of ovarian cancer have been controversially demonstrated. In this study, we profiled the phenotypes of mouse compact bone-derived MSCs (CB-MSCs) and bone marrow-derived MSCs (BM-MSCs) and found that CB-MSCs expressed lower CD90 compared to BM-MSCs. We examined gene expression of immune regulating cytokines of CB-MSCs in 2D and 3D culture and under stimulation with TLR4 agonist LPS or immune activator VIC-008. Our data showed that when CB-MSCs were cultured in simulated *in vivo* 3D condition, CD90 expression was further decreased. Moreover, gene expressions of immune activating cytokines *IL-12*, *IL-21*, *IFNγ* and a pro-inflammatory cytokine *CXCL10* in CB-MSCs were increased in 3D culture whereas gene expression of anti-inflammatory cytokines *IL-10* and *CCL5* were downregulated. Stimulation of CB-MSCs by LPS or VIC-008 presented similar profile of the cytokine gene expressions to that in 3D culture which might benefit the anti-tumor efficacy of CD90^low^ MSCs. The anti-tumor effects of CD90^low^ CB-MSCs alone or in combination with VIC-008 were evaluated in a syngeneic orthotopic mouse model of ovarian cancer. Treatment that combines CB-MSCs and VIC-008 significantly decreased tumor growth and prolonged mouse survival. This was associated with the increase of activated anti-tumoral CD4^+^ and CD8^+^ T cells and the decrease of Treg cells in the tumor microenvironment. Taken together, our study demonstrates the synergistic anti-tumoral efficacy by application of CB-MSCs combined with immune activator VIC-008 and provides new insight into CD90^low^ MSCs as a new anti-tumor arsenal.

## INTRODUCTION

Ovarian cancer is a life-threatening tumor in women as its diagnosis often occurs at a late stage [1]. In recent years, immunotherapy as adjuvant to surgery and chemotherapy has been broadly investigated in ovarian cancer as a way to reduce tumor recurrence and improve the survival rate [2]. Bone marrow derived mesenchymal stem/stromal cells (BM-MSCs) have also been studied for cancer therapy, due to their allogeneic availability, tropism for solid tumors, immune privilege and reliable *in vitro* amplification [3]. In the context of ovarian cancer, MSCs have been deployed as genetically engineered delivery vehicles, where they may highly express IL-2, IL-12, IL-15, IL-21, IFN-β or the suicide gene [4–6]. However, gene engineering has come along with safety concerns. Unmodified MSCs are seldom used to treat ovarian cancer since several studies have suggested that MSCs might promote tumor growth [7, 8]. As immunomodulatory cells, MSCs were mostly characterized with immunosuppressive properties. However, more emerging studies demonstrated the capability of MSCs in keeping immunity in balance [9]. In some circumstances, they could even promote immune priming. For instance, MSCs could be induced to act as antigen-presenting cells (APCs) [10, 11]. Approaches for polarizing MSCs into pro-inflammatory and anti-tumorigenic MSC1 or immunosuppressive and pro-tumorigenic MSC2 by TLR4 or TLR3 agonists were also reported [12, 13]. MSCs supported the survival of B cells through undetermined factors [14] and prevented T cells apoptosis through the secretion of IL-7 [15]. These studies suggest that MSCs may have the anti-tumor potential in tumor immunotherapy.

Ovarian cancer cells can form a highly inflammatory microenvironment contributing to the malignancy of the tumor. In particular, several pro-inflammatory cytokines such as TNF-α, IL-1β, and IL-6 produced by ovarian tumor cells or activated immune cells, have been shown to increase tumor growth and influence clinical disease outcome and prognosis [16]. MSCs have dominant advantages in inflammatory control and balance [9, 17]. Previous studies applied genetically engineered TRAIL-secreting MSCs to modulate inflammation and target tumors and showed significant effects in control of tumor burden, metastasis and tumor-associated inflammation in malignant glioma [18], colorectal carcinoma [19], metastatic renal cell carcinoma [20], pancreatic cancer [21] and mesothelioma [22, 23]. In the treatment of malignant mesothelioma, intraperitoneal administration of either MSCs alone or MSCs-TRAIL decreased the peritoneal inflammation by reducing levels of inflammatory cytokines IL-1α, CCL2 and TNF-α [22], indicating the inflammatory downregulation by MSCs in tumor microenvironment may also be applicable to the treatment of ovarian cancer.

The development of cancer immunotherapies favors combination strategies for enhancing APC function and enhancing T cell effector activity while eliminating immunosuppression [24]. Tumor-specific antigen targeting dendritic cell (DC) vaccines represent the most promising approach, which can orchestrate all of the elements of the immune system for cancer therapy [25]. Mesothelin is a tumor antigen that is highly expressed on the surface of common epithelial cancers including ovarian cancer [26]. Mesothelin expression appears to drive tumor growth and metastasis and is negatively associated with survival in ovarian cancer, which indicates it could be a therapeutic target. Previously, we developed a fusion protein consisting of anti-mesothelin scFv genetically fused with *Mycobacterium tuberculosis* heat shock protein 70 (Hsp70) (designated as VIC-008). The anti-mesothelin scFv provides tumor-specific targeting while Hsp70 facilitates both innate and adaptive immune responses toward the tumor. Immune activation by VIC-008 includes activating monocytes and DCs to produce CC-chemokines for attraction of antigen-processing and presenting DCs, macrophages, and effector T and B cells; enhancing DC aggregation and maturation; stimulating phagocytosis of distressed and foreign cells; and inducing cytotoxic activity of natural killer cells [27, 28]. Previous study has shown that VIC-008 primes an adaptive, tumor-specific CD8^+^ T cell immune response that is an absolute requirement for tumor control and survival prolongation in BR5FVB1 ovarian cancer mouse model [29]. The same finding was observed in a syngeneic orthotopic mouse model of ID8 ovarian cancer [30]. Although the fusion protein significantly enhanced survival of the ovarian tumor-bearing mice, it did not result in long-term remission. Our previous study also revealed that the increase of VIC-008 doses could further benefit mouse survival but could not delay onset of ascites and reduce ascites production, which may be the consequence of highly inflammatory microenvironment of malignant tumor.

To gain a better understanding of the potential for MSCs in cancer immunotherapy, in this study, we profiled the phenotypes of mouse compact bone derived MSCs (CB-MSCs) and found that CB-MSCs are different from bone marrow derived MSCs (BM-MSCs) in CD90 expression. Decreased expression of CD90 was reported to be associated with a loss of human MSC immunosuppressive activity [31], thus low CD90 expression observed in CB-MSCs might indicate a loss of immunosuppressive function, which might be helpful in the cancer immunotherapy. We further examined gene expression of immune regulating cytokines of CB-MSCs in 2D and 3D culture and under stimulation with VIC-008. Finally, we tested anti-tumor effects of unmodified CB-MSCs alone and in combination with VIC-008 and demonstrated the synergistic efficacy of the combination therapy.

## RESULTS

### CB-MSCs express lower CD90 and higher Sca-1 compared to BM-MSCs

**Figure 1:**
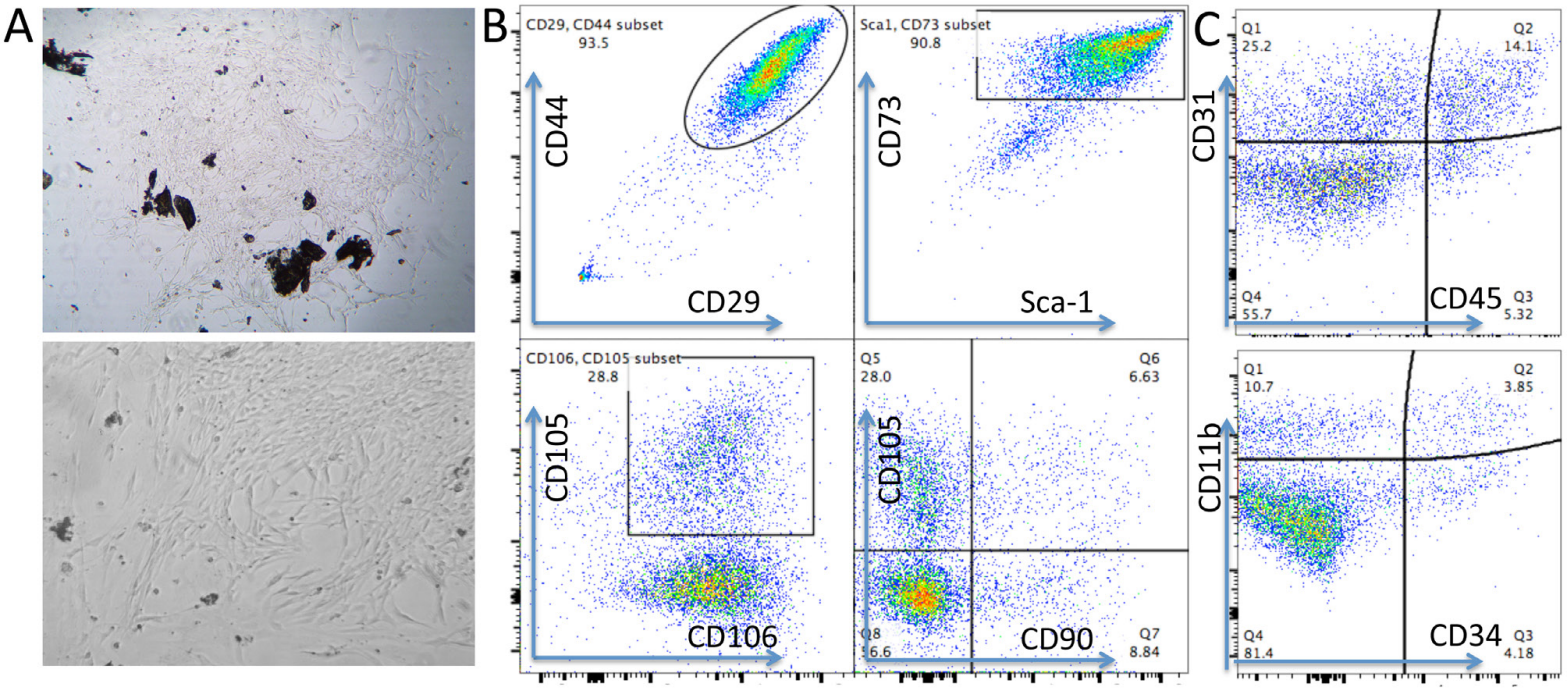
Phenotypes of mouse CB-MSCs. (**A**) Morphology of primary CB-MSCs under microscopy. Upper: 10 x magnification, Lower: 20 x magnification. (**B**) Representative cytometric dot plots. Phenotypic markers of CD29, CD44, Sca-1, CD73, CD105, CD106 and CD90 were stained to identify MSCs at passage 2. CD90 expressed very low on CB-MSCs. (**C**) Negative markers of CD45, CD31, CD34 and CD11b were stained to help identify MSCs.

**Figure 2:**
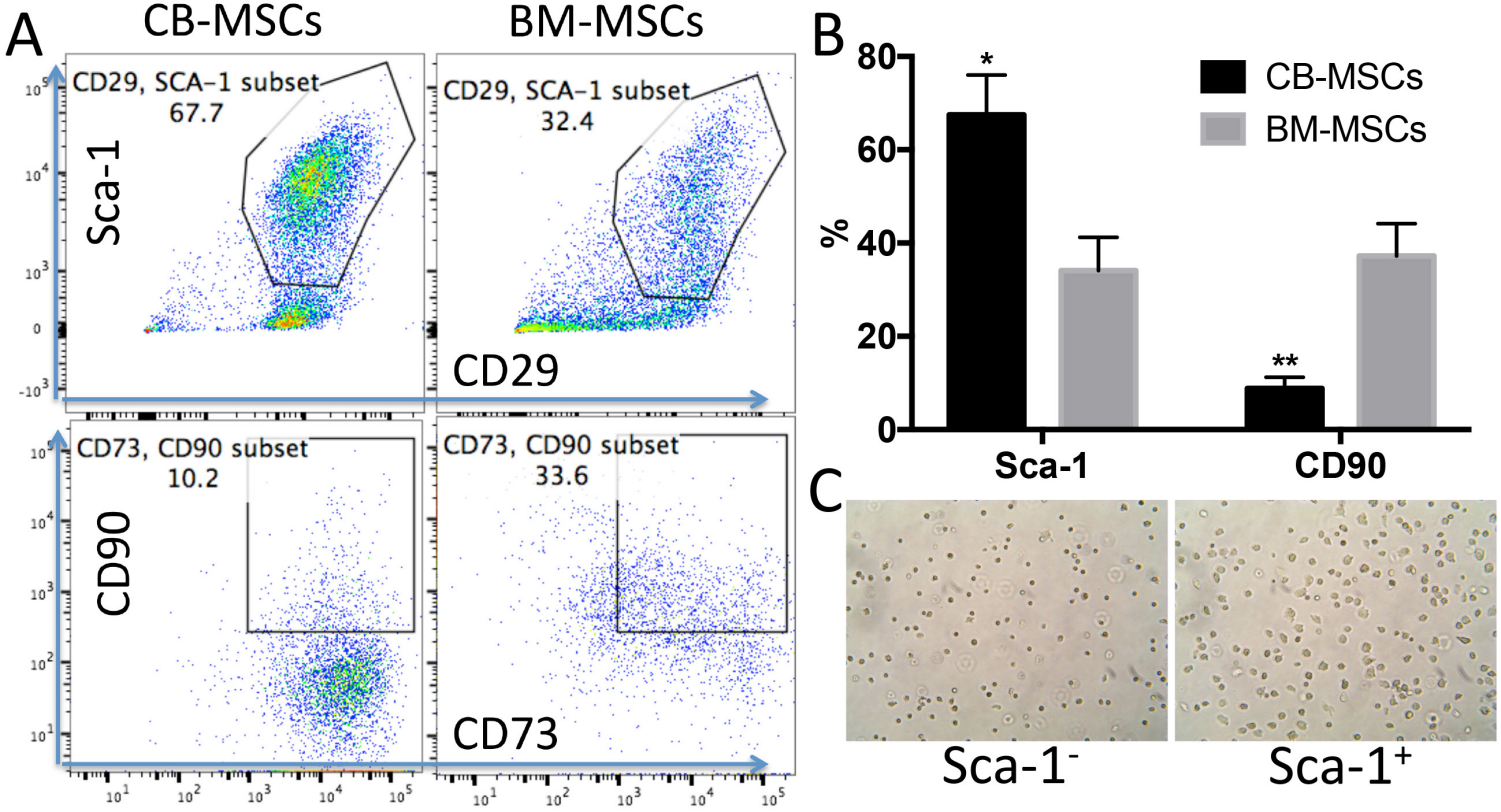
CB-MSCs express higher Sca-1 and lower CD90 compared to BM-MSCs. (**A**) Representative cytometric dot plots of MSCs at passage 6. (**B**) Percentage of Sca-1 and CD90 positive population in CB-MSCs and BM-MSCs. (**C**) Microscopic images of sorted Sca-1 negative (Left) and positive cells (right), 20 x magnification.

MSCs are heterogeneous population with different sources [32]. We isolated MSCs from compact bone. Most of the cells showed universe spindle like morphology (Figure 1A). However, cytometric results demonstrated the existence of distinct phenotypic subsets in the MSC population (Figure 1B, 1C). The cells highly express CD44, CD29, Sca-1, CD73 and CD106, and moderately express CD105 (Figure 1B). Notably, CD90, which is a marker for distinguishing human MSCs, was expressed at low frequency in mouse CB-MSCs (Figure 1B). This heterogeneous population also includes cells that express hematopoietic cell markers CD31, CD11b, CD45 and CD34 (Figure 1C). MHCII is not expressed (data not shown). We also collected BM-MSCs and compared with CB-MSCs. BM-MSCs expressed less Sca-1 than CB-MSCs and Sca-1 expression was further decreased in cells of passage 5–6 in both MSCs (Figure 2A, 2B). Within CD29^+^Sca-1^+^ population, CD90 expression is still low in CB-MSCs, but higher in BM-MSCs (Figure 2A, 2B). Sca-1^-^ and Sca-1^+^ populations showed distinct morphologies (Figure 2C), with Sca-1^-^ cells showing smaller and round shape but Sca-1^+^ cells showing bigger and stretched cell shape. CD90^+^ and CD90^-^ cells didn’t show obvious differences in morphology (data not shown). Decreased expression of CD90 was reported to be associated with a loss of human MSC immunosuppressive activity [31], thus low CD90 expression observed in CB-MSCs might indicate a loss of immunosuppressive function, thereby granting tumor-targeting T cells to execute tumor killing.

### CD90^low^ CB-MSCs inhibited tumor growth and prolonged survival

**Figure 3:**
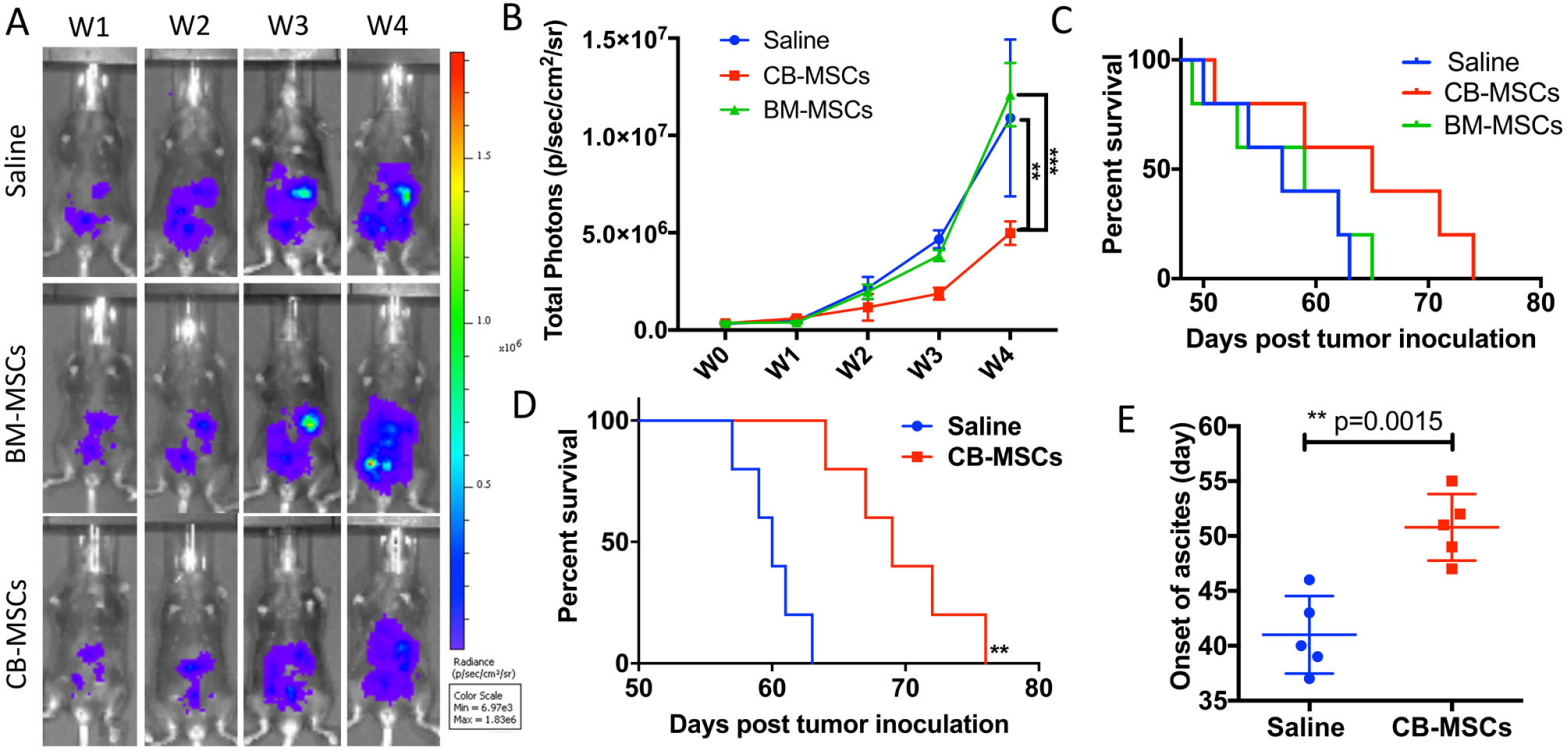
CD90^low^ CB-MSCs inhibited tumor growth and prolonged mouse survival in ovarian cancer model. (**A**) Representative bioluminescent images of CB-MSCs and BM-MSCs treated mice compared to saline treated group. MSCs were administered i.p. at week 0 and week 3 at the number of 3 × 10^6^ and 0.5 × 10^6^. (**B**) Quantified signal of luciferase-expressing ID8 tumor cells (*n* = 5). (**C**) Mice survival curve (*n* = 5). (**D**) Mouse survival when MSCs were administered i.p. at week 0 at the number of 3 × 10^6^ and the increased number of 3 × 10^6^ at week 3. (**E**) The time of onset of ascites in saline control and CB-MSCs applied groups.

In testing of anti-tumor effect of CB-MSCs and BM-MSCs in a mouse model of ID8 ovarian cancer, inhibition of tumor growth was observed in CB-MSCs group (Figure 3A and 3B). BM-MSCs treatment showed no difference in benefit of survival compared to saline control with median survival of 59 and 60 days respectively. However, the survival benefit was modest when 3 × 10^6^ CB-MSCs were applied at week 0 (i.e. 1 week after tumor cell inoculation) and 0.5 × 10^6^ CB-MSCs applied at week 3 (Figure 3C). When CB-MSCs was increased to 3 × 10^6^ for each treatment and applied every 3 weeks for twice (Figure 3D), the mouse survival was further significantly improved with median survival from 65 days to 69 days. Meanwhile, the onset of ascites was significantly delayed (Figure 3E). These data suggested that CB-MSCs might contain a different population that could slow tumor progression.

### Increased gene expressions of immune-activating cytokines and decreased gene expressions of immunosuppresive cytokines in CD90^low^ CB-MSCs

**Figure 4:**
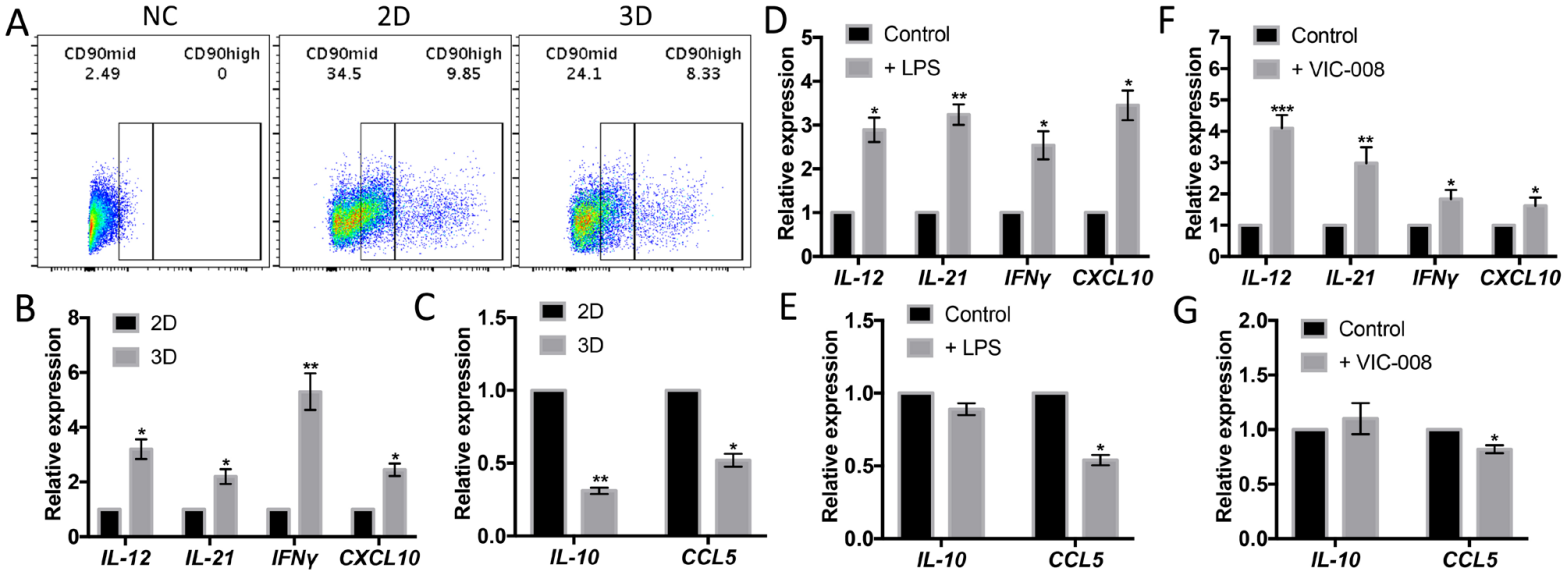
CD90^low^ CB-MSCs increased gene expression of immune-activating cytokines and decreased gene expression of immunosuppresive mediators. (**A**) CD90^high^ and CD90^mid^ populations from CB-MSCs were downregulated while cultured in 3D environment. (**B**) Gene expression of *IL-12*, *IL-21*, *IFNγ* and *CXCL10* in 2D and 3D culture quantitated by qRT-PCT. (**C**) Gene expression of *IL-10* and *CCL5* in 2D and 3D culture quantitated by qRT-PCT. (**D**) Gene expression of *IL-12*, *IL-21*, *IFNγ* and *CXCL10* in TLR-4 stimulated CB-MSCs. (**E**) Gene expression of *IL-10* and *CCL5* in TLR-4 stimulated CB-MSCs. (**F**) Gene expression of *IL-12*, *IL-21*, *IFNγ* and *CXCL10* in VIC-008 stimulated CB-MSCs. (**G**) Gene expression of *IL-10* and *CCL5* in VIC-008 stimulated CB-MSCs. *n* = 5.

While CB-MSCs cultured in 3D condition, which is a simulation of cell growth *in vivo*, CD90^mid^ and CD90^high^ populations were both decreased (Figure 4A). Gene expressions of immune-activating cytokines *IL-12*, *IL-21*, *IFN*γ and *CXCL10* were significantly increased (Figure 4B) whereas gene expressions immunosuppresive cytokines *IL-10* and *CCL5* were significantly decreased (Figure 4C). We also check the gene expressions of CB-MSCs upon TLR-4 stimulation with LPS and found that LPS had the similar impact on cytokine profile on CB-MSCs as in 3D culture (Figure 4D, 4E). VIC-008 contains immune-stimulating factor HSP70 that has been studied for ovarian cancer therapy in many ways [33–35]. By coculturing CB-MSCs with VIC-008, we found similar gene expression changes to TLR-4 stimulation, characterized by upregulation of *IL-12*, *IL-21*, *IFN*γ and *CXCL10* but downregulation of *CCL5* (Figure 4F, 4G). *IL-10* expression was not obviously changed under either TLR-4 or VIC-008 stimulation. These data further suggested that CB-MSCs might be stimulated and primed toward an immune-activating phenotype.

### Combination of CD90^low^ CB-MSCs with VIC-008 further improved anti-tumor efficacy

**Figure 5:**
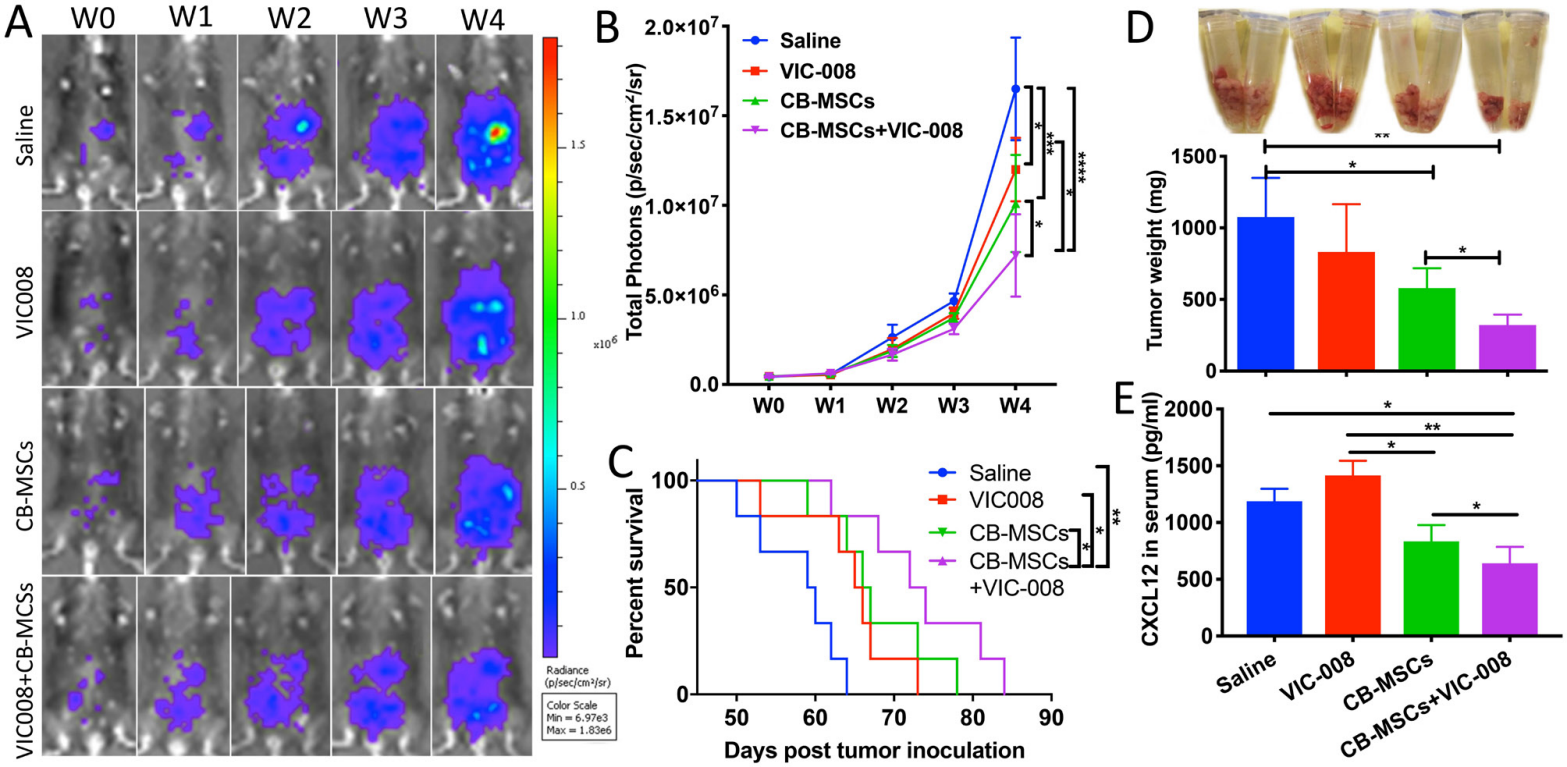
Combination of CD90^low^ CB-MSCs with VIC-008 further improved anti-tumor efficacy. (**A**) Representative bioluminescent images of different treatment groups. (**B**) Quantification signal of luciferase-expressing ID8 tumor (*n* = 6). (**C**) Mice survival curve (*n* = 6). (**D**) Tumor weight at week 7 post tumor inoculation *n* = 5. (**E**) Quantification of CXCL12 in the blood serum by ELISA. *n* = 5.

Next, CB-MSCs were applied in combination with VIC-008 for the treatment of ovarian cancer in a mouse model. The results showed that CB-MSCs or VIC-008 alone significantly inhibited tumor growth (Figure 5A, 5B) and extended animal survival (Figure 5C). The combination treatment significantly suppressed tumor growth and prolonged mouse survival to a median survival of 73 days in comparison to CB-MSCs or VIC-008 alone with median survival of 67 days and 66 days, respectively (Figure 5A–5C). In another set of experiment, we sacrificed the mice 7 weeks post treatment and collected tumor tissues. We weighed tumor mass and found CB-MSCs alone and the combination therapy significantly controlled tumor mass compared to saline control treatment (Figure 5D). We also found lowest amount of CXCL12 in the serum of combination treatment group (Figure 5E), which is in line with the previous finding that CXCL12 levels in serum are correlated with ovarian cancer prognosis [36]. VIC-008 monotherapy showed limited effects on tumor mass control and CXCL12 expression. These data suggested the synergistic anti-tumor efficacy of combination therapy.

### Combination therapy reduced Treg cells and activated CD8 T cell both in splenocytes and tumor microenvironment

**Figure 6:**
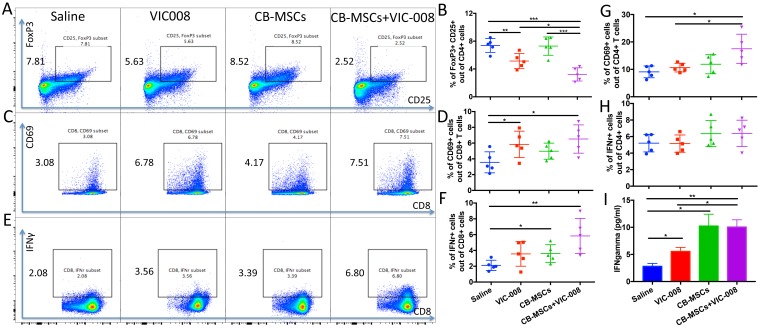
Combination therapy reduced Treg cells and activated CD8^+^ T cells and in splenocytes. (**A, B**) Representative cytometric dot plots and quantification of CD4^+^CD25^+^FoxP3^+^ Treg cells. (**C, D**) Representative cytometric dot plots and quantification of CD8^+^CD69^+^ T cells. (**E, F**) Representative cytometric dot plots and quantification of CD8^+^IFNγ^+^ T cells. (**G**) Percentage of CD4^+^CD69^+^ cells. (**H**) Percentage of CD4^+^IFNγ^+^ T cells. (**I**) Quantification of IFNγ in the supernatant of cultured splenocytes after stimulation by mesothelin peptides by ELISA (*n* = 5).

We further investigated immune responses in spleen from tumor-bearing mice at week 7 post treatment. The results showed that MSCs combined with VIC-008 significantly decreased the proportion of CD4^+^CD25^+^FoxP3^+^ Treg cells. VIC-008 alone also downregulated the CD4^+^CD25^+^FoxP3^+^ Treg cells but not CB-MSCs monotherapy (Figure 6A, 6B). CD8^+^ T cell activation marker CD69 was highly expressed on CD8^+^ T cells in the combination therapy group (Figure 6C, 6D). IFNγ^+^CD8^+^ T cell population was significantly increased in both CB-MSC monotherapy and combination treatment groups (Figure 6E, 6F). In combination therapy group, CD4^+^ T cells showed significantly increased CD69 expression and slightly increased IFNγ expression (Figure 6G, 6H). We cocultured isolated splenocyte with mesothelin peptides and found that VIC-008 moderately increased IFNγ in the supernatant; but there was a higher level of IFNγ in the supernatant in CB-MSCs applied groups (Figure 6I).

**Figure 7:**
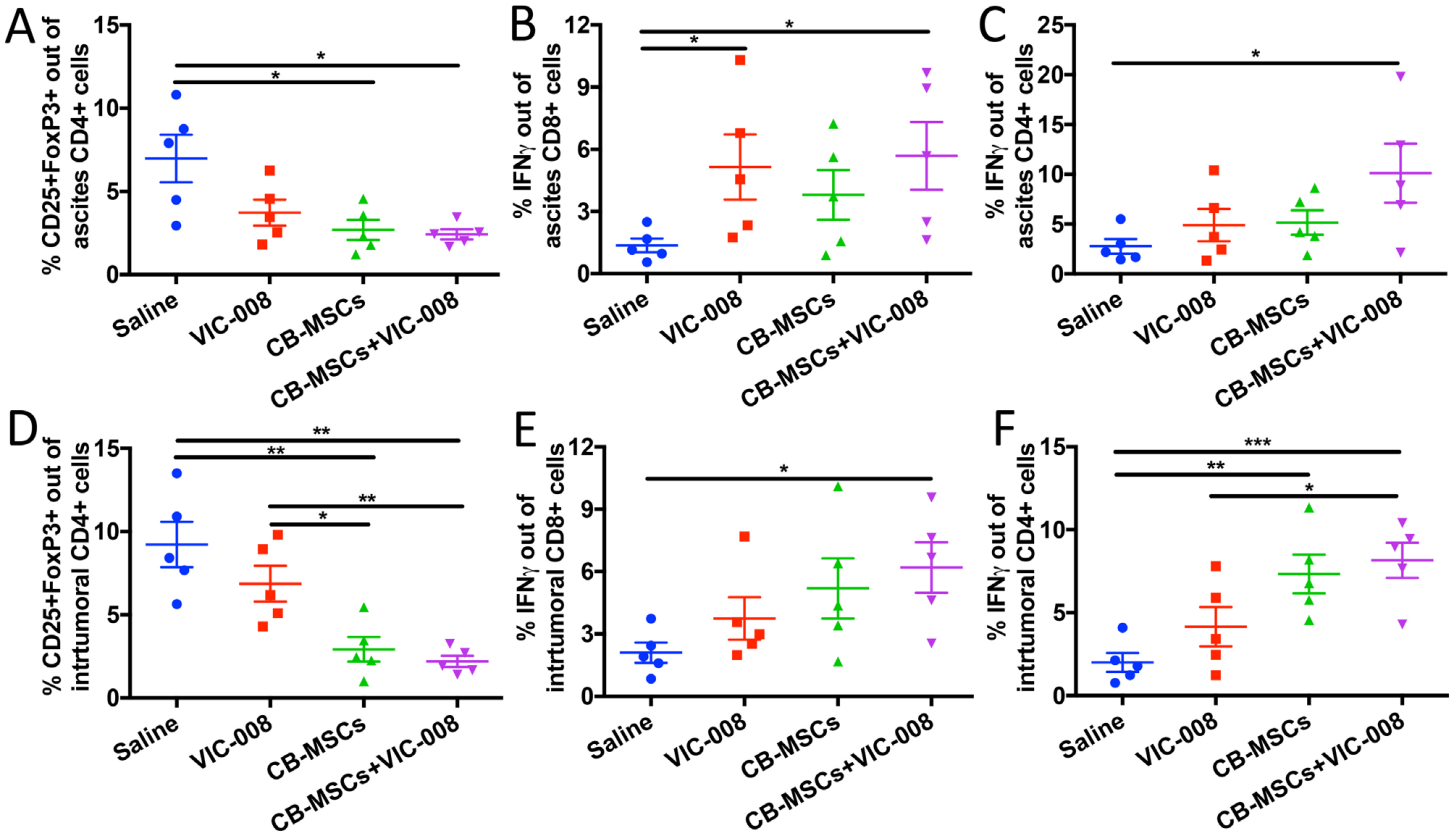
Combination therapy decreased Treg cells and increased CD8 activation in tumor environment. (**A**) Quantification of CD4^+^CD25^+^FoxP3^+^ Treg cells in ascites. (**B**) Quantification of CD8^+^IFNγ^+^ cells in ascites. (**C**) Quantification of CD4^+^IFNγ^+^ cells in ascites. (**D**) Quantification of intratumoral CD4^+^CD25^+^FoxP3^+^ Treg cells. (**E**) Quantification of intratumoral CD8^+^IFNγ^+^ T cells. (**F**) Quantification of intratumoral CD4^+^IFNγ^+^ T cells.

We also collected ascites and tumor tissue to profile immune responses therein. Treg cells were significantly decreased in CB-MSC monotherapy and combination treatment groups (Figure 7A, 7D). Activated CD8^+^IFNγ^+^ T cells were significantly increased in VIC-008 and combination groups in ascites (Figure 7B). In tumor, CD8^+^IFNγ^+^ cells were significantly increased in combination group only (Figure 7E). CD4^+^ IFNγ^+^ T cells were significantly increased in the combination group in ascites (Figure 7C). CD4^+^IFNγ^+^ T cells were elevated in tumor in both CB-MSC monotherapy and combination treatment groups (Figure 7F). Taken together, these data suggested that CB-MSCs greatly contributed to the immunotherapeutic efficacy.

## DISCUSSION

Although it was reported that MSCs could differentiate into carcinoma-associated fibroblasts (CFA) and pericytes to support tumor growth [37], an unique population of MSCs might have different fate. In tissue regeneration study, it is generally believed that MSC-based therapy not only provides a source of cells with which to regenerate a tissue but also regulates inflammation and ‘empowers’ other cells to facilitate tissue repair by the MSC secretome [38]. Published studies have shown that the therapeutic effect of MSCs is mainly a result of immunomodulation and that this function is ‘licensed’ by inflammation [39]. This indicates that MSCs could have a universal crosstalk with the pro-inflammatory factors and anti-inflammatory factors in tumor microenvironment. The anti-tumor activity of MSCs in tumor models such as hepatoma and breast cancer has also been shown to result in downregulation of Akt, NF-kB and Wnt signaling pathways in the tumor cells [3]. However, the immune regulation of MSCs for tumor-suppression was rarely elucidated. In current study, we found that compact bone derived MSCs showed lower CD90 expression compared to BM-MSCs, and this population significantly improved anti-tumoral effects while applied alone or in combination with a mesothelin-targeted and immune-activating fusion protein VIC-008.

Previous study provided evidence that hMSC with a significant decrease in CD90 surface molecule expression could elicit a lymphoproliferative allogeneic response in MSC/PBMC/PHA co-cultures [31]. sHLA-G and IL-10 concentrations culture supernatants were not increased. These results suggested that hMSCs with a depressed expression of CD90 exhibit a diminished immunosuppressive activity on T cell proliferation. Other studies showed CD90 expression might have immunosuppressive function. For instance, intraperitoneal CD90^+^CD45^-^ mesothelial cells markedly suppressed T cell proliferation with the reduction of CD3 ζ chain expression [40]. In another study, Coculture of Colonic CD90^+^ mesenchymal myofibroblasts and fibroblasts with resting or naïve CD4^+^ T cells led to development of cells with Treg phenotype [41]. Our results showed that CD90^low^ CB-MSCs could inhibit tumor growth and prolong mouse survival, whereas CD90^+^ BM-MSCs didn’t exhibit any anti-tumoral effects in our mouse model of ovarian cancer. These data indicated that CD90 might play an important role in the immune modulation for cancer therapy. MSCs are heterogeneous cell population which could be derived from different tissues and have multilineage differentiation potential. Sca-1 is a known stem cell marker. In this study we found that Sca-1^-^ and Sca-1^+^ MSCs show distinct morphologies and that CB-MSCs express lower CD90 and higher Sca-1 compared to BM-MSCs. This is associated with superior antitumor efficacy of CB-MSCs to BM-MSCs. More work is needed in the future to determine if defined phenotypes based on CD90 and Sca-1 in MSC population could predict different antitumor activity.

In this study, several genes related to immune regulation that is not mainly expressed in MSCs, were significantly upregulated or downregulated in CB-MSCs under several conditions. These results are in line with a panel of previous findings. For example, MSCs were reported to be engineered to highly express IL-12 [42], IL-21 [43] or IFNγ [44] to enhance tumor targeting. CXCL10, a pro-inflammatory cytokine, was increased dramatically while MSCs cultured in macrophage-conditioned medium [45]. IL-10 is an anti-inflammatory cytokine that could inhibit synthesis of pro-inflammatory cytokines and downregulate immune activation [46]. CCL5-CCR5 interaction was reported to be related to MSC-induced tumor metastasis [47, 48]. Other than 3D condition, both TLR-4 stimulation and HSP70 contained VIC-008 coculture stimulated similar pattern of gene expression in CB-MSCs. TLR-4 stimulation can polarize MSCs into pro-inflammatory and anti-tumorigenic MSC1 [12, 13]. HSP70 engineered MSCs were validated in enhanced treatment of myocardial damage [49]. A recent study also reported that recombinant HSP70 and mild heat shock could stimulate growth of aged MSCs [50]. All these evidences indicated that CD90^low^ CB-MSCs might be tuned into macrophage-like antigen presenting cells and possess an immune-activating property.

It has been recently reported that MSCs could undergo hematopoietic differentiation and achieve a macrophage-like phenotype in tumor progression [51]. MSCs may be stimulated to act as APCs [52]. The contribution of myeloid origin stromal cells to immune escape in tumor microenvironment has been intensively studied in the last decade, represented by myeloid-derived suppressor cells (MDSCs) [53], tumor-associated M2 macrophages (TAMs) [54], and regulatory dendritic cells (DCs) [55]. The infiltrated portions of these cells have great influence on tumor progression [56, 57]. VIC-008 has been verified to stimulate DC maturation and cross-presentation of tumor antigens and might also induce MSCs into immune-activating cells in the presence of tumor antigens. Our results showed improved IFNγ secretion upon stimulation with mesothelin peptide in VIC-008 treated group. CB-MSCs applied groups showed increase of IFNγ secretion, which indicates that CB-MSCs themselves might also increased antigen presentation to T cells. Combination of CB-MSCs with VIC-008 further significantly increased intratumoral CD8^+^IFNγ^+^ T cell population, which suggested synergistic effects of the combination therapy.

Although we showed promising results of anti-tumoral effects of CD90^low^ CB-MSCs alone and in combination with VIC-008, our study still has some limitations which need to be studied in the future. In this study, we used VIC-008 at 10 μg/100 μl per mouse while in some other studies we used 20 μg/100 μl per mouse that improved the therapeutic efficacy. Dosage of CB-MSCs may be further increased to benefit the survival as well. Since some studies suggested that MSCs might promote tumor metastasis, more work is needed to exclude the possibility of CD90^low^ MSC-promoted tumor metastasis. The fate of MSCs should also be further explored in the future to better understand their roles in tumor microenvironment. It should also be good to screen and purify CD90^low^ MSC population, and further detect markers indicated in other research for subpopulation identification, such as CD146 (a marker related to supporting hematopoiesis) [58, 59], LepR (a marker related to enhanced osteogenic differentiation) [60] and CXCR4 (a marker related to cell migration and engraftment) [61]. More work is needed to systematically profile transcriptome of CD90^low^ and CD90^high^ MSCs by RNA-Seq analysis. More work is also needed to compare CD90^low^ with CD90^high^ MSCs in study of immunomodulatory effects of the MSCs by co-culturing with different lymphocytes and observing the lymphocyte proliferation and activation. Profiling of cytokine expression of CD90^low^ and CD90^high^ subpopulation will be our next effort to determine selection of right types of MSCs for different therapeutic needs in the future.

In summary, we found the difference of CD90 expression in CB-MSCs and BM-MSCs and evaluated the anti-tumoral effects of unmodified cells in mouse model of ID8 ovarian cancer. Our data demonstrated the synergistic anti-tumoral efficacy by application of CB-MSCs combined with fusion protein VIC-008 and revealed mechanisms by which CB-MSCs modulates immunity alone and in combination with VIC-008. Taken together, our study for the first time identifies an anti-tumoral subtype of MSCs and provides new mechanistic insight into CB-MSC-mediated immunomodulation and highlight the enhanced antitumor efficacy of a cell therapy alone and in combination with a tumor antigen-targeted therapy in mouse ovarian cancer, which could be clinically relevant to patients with this disease.

## MATERIALS AND METHODS

### Isolation of MSCs

MSCs were isolated based on a protocol from Stem Cell Company (https://www.stemcell.com/mesencult-expansion-kit-mouse.html). Briefly, the femurs and tibias were obtained from C57BL/6J female mice (4–6 weeks) under aseptic operation. Muscle and connective tissues were totally removed. A syringe pestle was applied to lightly crush the bones to release the marrow. The marrow was filtered and collected for BM-MSCs culture. To generate CB-MSCs, the crushed bone was cut into 1–2 mm pieces and digested by 0.25% Collagenase Type I for 45 min. 10 ml buffer containing PBS, 2% FBS and 1 mM EDTA was used to wash and collect the digested cells. Subject to centrifuge, the cell pellet from bone marrow or the cell pellet and bone fragments are cultured in complete MesenCult™ Medium (StemCell, # 05513) with MesenPure in T-75 tissue culture flasks. Cell cultures were maintained at 37°C in a humidified incubator with 5% CO_2_. Cells were passaged at the ratio of 1:3 at confluent. Cells of passage 2 to 6 were used in following experiments. The stem cell markers of each passage were examined.

### Flow cytometry

MSC and splenocyte suspensions were washed with PBS and stained with Zombie UV viability dye (BioLegend, #423108) and then washed with cell staining buffer (BioLegend, #420201). The cells were then stained with multiple fluorophores conjugated cell surface antibodies (Supplementary Table 1). Fixation/permeabilization reagents was applied for intracellular staining (BioLegend, #424401). Cells were then stained with fluorophores conjugated intracellular antibodies. The compensations were set up before running the stained cells on Fortessa X-20 (BD Biosciences). Storage events were gated on interested population. Flow data was analyzed by FlowJo V10.

### 3D culture of CB-MSCs

Gelatin microcryogels (GMs) was kindly provided by Du lab in Tsinghua University. The fabrication and use was fully elucidated as previously described [38, 62]. In this study, CB-MSCs were seeded into GMs at the density of 1 × 10^7^ cells/ml. The cells suspension was dropped on the monolayer of GMs for auto-absorbing. After incubation in humidified 37°C incubator for 1.5 h for cell attachment, cell-laden GMs were transferred into a new dish and culture medium was added. Two-day culture was applied before cellular analysis.

### Stimulation of CB-MSCs by LPS and VIC-008

LPS were purchased from Sigma-Aldrich as agonist for TLR-4. The fusion proteins VIC-008 were constructed as described previously [29] and expressed by WuXi App Tech (Shanghai, China) in CHO cells and provided at a purity of above 95% by HPLC and an endotoxin level of less than 1.0 EU/mg. For coculture, LPS was applied at 10 ng/ml for 24 hours; VIC-008 was applied at 5 ug/ml for 48 hours.

### Real-time PCR

Cultured cells were lysed by TRIzol (Invitrogen, USA) and RNA isolation and reverse transcription were performed as previously described [63]. Real Time PCR amplification was performed in triplicates according to procedures reported previously. Relative expression of mRNA was evaluated by 2^-ddct^ method and normalized to the expression of β-actin. The primer of the related genes lists in Table 1.

**Table 1:** Primers used in this study

Gene primers	Sequences	Length
β-actin forward	AGAGGGAAATCGTGCGTGAC	20
β-actin reverse	CAATAGTGATGACCTGGCCGT	21
*IL-12* forward	CTGTGCCTTGGTAGCATCTATG	22
*IL-12* reverse	GCAGAGTCTCGCCATTATGATTC	23
*IL-21* forward	GGACCCTTGTCTGTCTGGTAG	21
*IL-21* reverse	TGTGGAGCTGATAGAAGTTCAGG	23
*IFNγ* forward	TCAAGTGGCATAGATGTGGAAGAA	24
*IFNγ* reverse	TGGCTCTGCAGGATTTTCATG	21
*CXCL10* forward	CCAAGTGCTGCCGTCATTTTC	21
*CXCL10* reverse	GGCTCGCAGGGATGATTTCAA	21
*IL-10* forward	GCTCTTACTGACTGGCATGAG	21
*IL-10* reverse	CGCAGCTCTAGGAGCATGTG	20
*CCL5* forward	CAGCAGCAAGTGCTCCAATCTT	22
*CCL5* reverse	TTCTTGAACCCACTTCTTCTCTGG	24

### Tumor cells

Luciferase-expression epithelial ovarian cancer cell line ID8 were previously generated in the lab. Before injection into mice, the cells were cultured in T75 flasks at 37°C in DMEM supplemented with 10% heat-inactivated fetal bovine serum (FBS; Thermo Fisher Scientific), 100U/mL penicillin and streptomycin (Mediatech), 2 mM L-glutamine (Thermo Fisher Scientific). Female C57BL/6J mice injected intraperitoneally (i.p.) with syngeneic mouse ID8 cells would develop clinically evident intra-abdominal tumors [64]. The ID8 cells formed direct contact with the ovarian stroma, resulting in primary tumor formation, secondary peritoneal carcinomatosis, and extensive ascites at late stage [65]. The cytological and architectural features resembled serous carcinoma.

### Animal model and treatment

C57BL/6J female mice (4–6 weeks) were purchased from the Jackson Laboratory and maintained in the MGH animal facility. After one-week accommodation in the animal facility, 5 × 10^6^ ID8-luc cells were administered i.p. per mouse. The establishment of the tumor model was identified by positive signal (total photons > 1 × 10^5^) using IVIS bioluminescent imaging one week after tumor cell inoculation. All tumor-bearing mice were randomly divided into different groups (*n* = 5 to 6 in each group) and received saline, CB-MSCs, BM-MSCs, VIC-008 or CB-MSCs + VIC-008 treatments. VIC-008 were applied ip. weekly for 4 weeks form week 1 at the dose of 10 μg/100 μl per mouse. For anesthesia when needed, the mice were administrated ip. with Ketamine (9 mg/ml in saline) and Xylazine (0.9 mg/ml in saline) at 0.1 ml per 10 g of body weight. All animal studies were approved by the Institutional Animal Care and Use Committee of Massachusetts General Hospital.

### Tumor growth and mouse survival

Tumor growth was evaluated by IVIS (PerkinElmer) each week post tumor cell inoculation. Luciferin (Regis Technologies, #1-360222) was applied at the concentration of 30 mg/ml. The mice received 100 μl Luciferin ip. 5 min before imaging. Total photons under the region of interest (ROI) captured by IVIS were used for statistical analysis. Survival time was calculated as life span from the day of tumor inoculation. In some cases, MGH CCM faculties will evaluate severe midsection swelling of the mice and poor body condition, and the life spans of the mice were recorded at the day of euthanasia.

### Processing of splenocytes and stimulation *ex-vivo*

Spleens were harvested at 7 weeks post tumor inoculation and placed in cold PBS and then mashed by two baked frosted glass slides. Splenocytes were passed through 40 μm cell strainers to get rid of connective tissue and then lysed by RBC lysis buffer. 2 × 10^6^ splenocytes were placed per well in 24-well plates cultured with RPMI 1640 medium supplemented with L-glutamine and FBS. 2 μg/ml recombinant mouse mesothelin (BioLegend, #594006) was applied for 72-hour stimulation. Brefeldin A and Monesin (BioLegend, #420601 and #420701) were added into the culture medium during the last five hours. The supernatant was collected to detect IFNγ by ELISA. The splenocytes were then harvested and prepared for flow cytometry.

### ELISA

Supernatant IFNγ and serum CXCL12 were evaluated by Duoset ELISA kit purchased from RandD Systems. For CXCL12, blood was drawn and serum was extracted 6 weeks post tumor inoculation. Following the manufacturer’s instructions, three times diluted serum was reacted with capture antibody and detection antibody. Microplate reader read the 96-well plates at 450 nm.

### Processing of ascites and ascites cells

7 weeks post tumor inoculation the mice were sacrificed by CO2 and processed to obtain the ascites and tumors. Ascites was centrifuged at 1500 rpm for 5 min to get rid of supernatant. Red blood cells (RBCs) were lysed by RBC lysis buffer (BioLegend, #420301) twice. The cells were then immunostained for flow cytometric analysis. Tumor tissues were sliced into 1–2 mm pieces and dissociated in DMEM supplemented with 2 mg/ml collagenase type IV (ThermoFisher, #17104-019), 2 mg/ml hyaluronidase (Sigma, #H3506), and 2 mg/ml BSA (Sigma, #05470) for 1.5 h at 37°C, shaking at a very slow speed for digestion. The cell suspension was then filtered through 70 μm cell strainers to collect the cells for immunostaining.

### Statistical analyses

One-way ANOVA, two-way ANOVA and student *T*-test were applied for most statistical analysis. GraphPad Prism 7 was used to make graphs and calculate *P* values. Survival curves were analyzed by Kaplan-Meier method and log-rank test. *P* value ≤ 0.05 was considered statistic significant. ‘^*^’ refers to *P* ≤ 0.05; ‘^**^’ refers to *P* ≤ 0.01; ‘^***^’ refers to *P* ≤ 0.001, ‘^****^’ refers to *P* ≤ 0.0001. Error bars represented mean and SD or SEM.

## SUPPLEMENTARY MATERIALS



## Abbreviations

MSCs: mesenchymal stem cells
CB-MSCs: compact bone-derived mesenchymal stem cells
BM-MSCs: bone marrow-derived MSCs
APCs: antigen-presenting cells
TLR: toll-like receptor
TRAIL: tumor necrosis factor-related apoptosis-inducing ligand
DCs: dendritic cells
GMs: gelatin microcryogels
LPS: lipopolysaccharide
PCR: polymerase chain reaction
ELISA: enzyme-linked immunosorbent assay
Sca-1: stem cell antigen-1
HSP70: 70 kilodalton heat shock proteins
FoxP3: forkhead box P3
Tregs: regulatory T cells
MDSCs: myeloid-derived suppressor cells
TAMs: tumor-associated M2 macrophages
LepR: leptin receptor
