# Supplementary material for: CD90low MSCs modulate intratumoral immunity to confer antitumor activity in a mouse model of ovarian cancer
Source: Oncotarget. 2019 Jul 9;10(43):4479–91. doi: 10.18632/oncotarget.27065 (PMC6633895; doi:10.18632/oncotarget.27065)
Supplement: Supplementary file 1 [file oncotarget-10-4479-s001.pdf]

CD90^{low} MSCs modulate intratumoral immunity to confer antitumor activity in a mouse model of ovarian cancer

SUPPLEMENTARY MATERIALS

Supplementary Table 1: Antibodies used in this study

Antibody	Fluorophore	Clone	Company	Catalog Number
CD29	AF488	HMβ1-1	BioLegend	102211
CD44	APC	IM7	BioLegend	103011
CD73	PE	TY/11.8	BioLegend	127205
CD90	APC/Cy7	30-H12	BioLegend	105327
CD105	PE/Cy7	MJ7/18	BioLegend	120409
CD106	PerCP/Cy 5.5	429 (MVCAM.A)	BioLegend	105715
Sca1	BV421	D7	BioLegend	108127
MHC II	BV711	M5/114.15.2	BioLegend	107643
CD31	PE/Dazzle 594	390	BioLegend	102429
CD34	PE/Cy5	MEC14.7	BioLegend	119311
CD45	AF700	30-F11	BioLegend	103127
CD11b	BV605	M1/70	BioLegend	101237
CD3	BV421	17A2	BioLegend	100227
CD4	AF647	GK1.5	BioLegend	100426
CD8	PE/CY7	53-6.7	BioLegend	100721
CD25	APC	PC61	BioLegend	102012
FoxP3	PE	MF-14	BioLegend	126403
IFNγ	AF488	XMG1.2	BioLegend	505815
CD69	AF647	H1.2F3	BioLegend	104518
